# Anemia is associated with the risk of Crohn’s disease, not ulcerative colitis: A nationwide population-based cohort study

**DOI:** 10.1371/journal.pone.0238244

**Published:** 2020-09-08

**Authors:** Eun Ae Kang, Jaeyoung Chun, Jong Pil Im, Hyun Jung Lee, Kyungdo Han, Hosim Soh, Seona Park, Joo Sung Kim

**Affiliations:** 1 Department of Internal Medicine and Liver Research Institute, Seoul National University College of Medicine, Seoul, South Korea; 2 Department of Internal Medicine and Institute of Gastroenterology, Yonsei University College of Medicine, Seoul, South Korea; 3 Department of Internal Medicine, Gangnam Severance Hospital, Yonsei University College of Medicine, Seoul, South Korea; 4 Department of Statistics and Actuarial Science, Soongsil University, Seoul, South Korea; Copenhagen University Hospital Holbæk, DENMARK

## Abstract

Anemia is a common manifestation of inflammatory bowel disease (IBD), but it remains unclear whether anemia is associated with the development of IBD. We assessed the risk of developing IBD in anemic patients, and stratified the results with respect to their hemoglobin concentrations. A population-based study was conducted using the National Healthcare Insurance Service database in South Korea. We included individuals over 20 years’ old who participated in the national health screening program in 2009 (*n* = 9,962,064). Anemia was defined as a hemoglobin level less than 13 g/dL in men and less than 12 g/dL in women. We compared the rate of newly diagnosed IBD in anemic patients and non-anemic individuals. Newly diagnosed IBD was identified using both the ICD-10 medical code and specialized V codes for rare intractable diseases in South Korea. During the mean follow-up period of 7.3 years, the incidences of CD and UC in anemic patients were 2.89 and 6.88 per 100,000 person-years, respectively. The risk of CD was significantly higher in anemic patients than in non-anemic individuals [adjusted hazard ratio (aHR), 2.084; 95% confidence interval (CI), 1.769–2.455]. The risk of CD development was inversely proportional to the hemoglobin concentration. A J-curve relationship was observed between age and the risk of CD in anemic patients. The risk of CD in male anemic patients was significantly higher than that in female anemic patients (aHR, 1.432 vs. 1.240, respectively). By contrast, there was no statistically significant difference in the risk of developing UC in anemic and non-anemic individuals (aHR, 0.972; 95% CI, 0.880–1.073). This work indicates that anemia is related to the development of CD, and this risk was inversely proportional to the hemoglobin concentration.

## Introduction

Anemia is defined as a hemoglobin concentration less than 13 g/dL in men and less than 12 g/dL in women according to World Health Organization (WHO) standards [1]. Anemia can be caused by a variety of etiologies such as iron deficiency, chronic diseases, vitamin B_12_ or folate deficiency, and genetic factors. Anemia is a known risk factor for cardiovascular diseases, dementia, and chronic medical illness, leading to an increase in all-cause mortality [2–5].

Inflammatory bowel disease (IBD) includes Crohn’s disease (CD) and ulcerative colitis (UC). IBD is a chronic inflammatory gastrointestinal disorder that involves complex interactions among genetic, environmental, immunological, and microbiological factors. The incidence of IBD in South Korea has been increasing, which causes higher medical and socioeconomic burdens [6]. IBD patients experience various systemic manifestations related to intestinal inflammation during disease progression. Anemia is a prevalent extraintestinal manifestation of IBD, especially for patients with CD. Malabsorption, malnutrition, and micronutrient deficiency cause anemia in patients with IBD. Gastrointestinal bleeding also can induce or aggravate anemia [7, 8].

It is important to find a surrogate marker for early detection of IBD to prevent complications and surgery and improve the quality of life for those with IBD. Colonoscopy is the most effective diagnostic test to evaluate the presence of colitis in asymptomatic individuals, but this test is not appropriate for disease screening due to its expense and invasiveness. The fecal calprotectin is a useful surrogate marker for predicting clinical recurrence of IBD [9]. However, it remains to be determined whether fecal calprotectin can be used as a screening tool for the early detection of IBD [10, 11]. Anemia may be a promising surrogate marker for early detection of IBD, but it remains to be determined whether anemia can be a marker for the detection of subclinical IBD. Recently, we reported that anemia was significantly associated with an increased risk of CD over the age of 40 [12]. However, the impacts of anemia on the occurrence of CD based on the severity of anemia and various subgroups have not been fully evaluated yet. The aim of this study was to investigate the incidence and risk of IBD based on the presence and severity of anemia.

## Materials and methods

### Study design

This was a nationwide population-based cohort study using the National Health Insurance (NHI) database in South Korea. The NHI is a public health insurance that is mandatory for 97% of the Korean population, except for those with the lowest incomes who are supported by Medical aid. The National Health Screening Program (NHSP) is recommended every two years for adults over 20 years old. The NHI and NHSP database includes demographic data such as age, sex, residence, smoking, alcohol consumption, height, body weight, laboratory results, medical diagnoses based on the International Classification of Disease 10^th^ revision (ICD-10) codes, and drug prescriptions. As the burden of medical expenses for rare and incurable diseases has been steadily increasing, a specialized V code was developed in Korea for rare and intractable disease (RID) such as IBD. Physicians can register the V codes according to the diagnostic criteria for each RID.

### Study population

We included individuals over 20 years old who participated in the NHSP from 1^st^ of January 2009 to 31^st^ of December 2009, the index year. Patients diagnosed with IBD from 1^st^ of January 2002 to the index date were excluded from this study (washout period). Individuals who were diagnosed with IBD for the first year since 2009 were excluded to eliminate the possibility of IBD unrelated to anemia (lag period). We compared individuals with anemia to those who did not have anemia. The included population was followed until December 31, 2016.

### Definition and covariates

IBD was detected using both the ICD-10 codes (K50 for CD and K51 for UC) and the V codes for RIDs (V130 for CD and V131 for UC). The operational definition of IBD was validated as described previously [13–15], with a sensitivity of 94.5% for CD and 96.4% for UC. Anemia was defined based on WHO criteria as a hemoglobin level less than 13 g/dL for men and less than 12 g/dL for women. Anemia during follow-up was defined as the presence of anemia at a 2-year follow-up exam in 2011 but without anemia at baseline, 2009. The study participants were classified as current or former smokers or nonsmokers based on self-reporting. Current smokers were defined as persons who continued to smoke at least a total of 100 cigarettes lifelong, and former smokers were defined as those who had smoked more than a total of 100 cigarettes but quit smoking at least one month before enrollment. Nonsmokers were defined as those who had smoked less than 100 cigarettes. Alcohol consumption was categorized as non-drinkers, mild drinkers (alcohol consumption < 30 g/day), and heavy drinkers (alcohol consumption ≥ 30 g/day). BMI (kg/m^2^) was classified as follows: < 18.5 for underweight, 18.5–22.9 for normal-weight, 23.0–24.9 for overweight (pre-obese), and ≥ 25.0 for obese [16]. Physical activity was classified based on the intensity and frequency of exercise as follows: none, less than 2 times per week, and 2 times or more per week for at least 20 minutes [17]. Hypertension was defined as systolic/diastolic blood pressure ≥ 140/90 mmHg, or a combination of ICD-10 codes (I10–13, I15) and prescription for anti-hypertensive medications. Dyslipidemia was identified as an ICD-10 code (E78) and medications for dyslipidemia, or a fasting total serum cholesterol level of ≥ 240 mg/dL. Diabetes mellitus (DM) was identified using ICD-10 codes (E11–14) and anti-diabetic medications, or a fasting glucose level of ≥ 126 mg/dL. Metabolic syndrome was defined as a disease combined with hypertension, hyperglycemia, central obesity, and dyslipidemia based on the National Cholesterol Education Program Expert Panel and Adult Treatment Panel III criteria. Chronic kidney disease (CKD) was defined as a glomerular filtration rate (GFR) < 60 mL/min/1.73 m^2^, as estimated by the Modification of Diet in Renal Disease methods.

### Aims

The purpose of this study was to determine the risk of CD and UC based on the presence of anemia and hemoglobin levels to analyze whether anemia defined by WHO criteria could be used as a marker for the detection of subclinical CD and UC.

### Statistical analysis

Continuous variables were reported as mean ± standard deviation using Student’s *t*-test. Categorical variables were analyzed using the χ^2^ test. The incidence was determined as incidence cases divided by the summation of follow-up period and multiplied by 10^5^. Follow-up durations were expressed as person-years. Kaplan-Meier method was used to compare the cumulative incidence probability of UC and CD based on the presence of anemia. The hazard ratio (HR) of the risk for developing IBD was analyzed with 95% confidence intervals (CI) using the Cox proportional hazard method. The adjusted HRs for age, sex, BMI, smoking, alcohol consumption, physical activity, metabolic syndrome, income, and GFR were calculated. Subgroup analysis was performed with respect to age, sex, CKD, overweight, and smoking. A *P* value < 0.05 was considered as statistically significant. All statistical analyses were conducted using SPSS version 20.0 (SPSS Inc., Chicago, IL, USA) and SAS Version 9.3 (SAS Institute, Cary, NC, USA).

### Ethical considerations

The Institutional Review Board (IRB) of the Seoul National University Hospital approved the study waived the need for informed consent as part of my study approval due to the non-interventional, retrospective design of the study (IRB Number, H-1703-107-840). All resident registration numbers were encrypted to protect personal privacy. All data used in this analysis was completely anonymous and all personally identifiable information was removed. The public health data could be obtained through the approval of Korea NHI data sharing service (NHIS-2019-1-155).

## Results

### Baseline characteristics of study population

A total of 9,962,064 people who participated in the NHSP during the index year were included in this study. Among them, 1,095,924 individuals (11%) had anemia according to WHO criteria. The mean ages of anemic and non-anemic participants were 51.1 and 46.6 years, respectively. Mean follow-up period was 7.3 ± 0.6 years. Compared to individuals without anemia, those with anemia were significantly associated with old age, female gender, non-smokers, non-drinkers, low physical activity, low BMI, low income, and urban residence (*P*-value for each variable < 0.0001). The prevalence of hypertension and DM was significantly higher in participants with anemia than in those without anemia (*P*-value for each variable < 0.0001). By contrast, dyslipidemia and metabolic syndrome were significantly less common in the anemic group than in the non-anemic group (*P*-value for each variable < 0.0001). Participants with anemia showed significantly lower serum creatinine levels and GFR than those without anemia (Table 1).

**Table 1:** Baseline characteristics of the study population.

	Anemia ^a^		
No. (%)	No	Yes	*P-*value
No. of patients	8,866,140	1,095,924	
Age, years ^b^	46.64 ± 13.88	51.13 ± 15.22	< .0001
Male (%)	5,197,461 (58.6)	256,831 (23.4)	< .0001
Height, cm ^b^	164.51 ± 9.19	158.82 ± 7.78	< .0001
Body weight, kg ^b^	64.69 ± 11.60	57.73 ± 9.37	< .0001
BMI, kg/m^2^ ^b^	23.81 ± 3.20	22.86 ± 3.12	< .0001
< 18.5 (underweight)	312,457 (3.5)	65,809 (6.0)	
18.5–23.0 (normal)	3,380,556 (38.1)	541,315(49.4)	
23.0–25.0 (overweight)	2,224,838 (25.1)	242,013(22.1)	
25.0–30.0 (obese I)	2,628,311 (29.7)	222,445(20.3)	
> 30.0 (obese II)	319,978 (3.6)	24,342(2.2)	
Waist circumference, cm ^b^	80.63 ± 9.01	77.00 ± 8.90	< .0001
Residence; Urban (%)	4,078,434 (46.0)	507,467 (46.3)	< .0001
Income (%) ^c^	811,322 (10.28)	284,602 (13.77)	< .0001
Smoking (%)			< .0001
Nonsmoker	5,023,316 (56.7)	904,726 (82.6)	
Former smoker	1,340,490 (15.1)	87,931 (8.0)	
Current smoker	2,502,334(28.2)	103,267 (9.4)	
Drinking (%)			< .0001
None	4,357,263 (49.1)	775,320 (70.7)	
Mild	3,855,113 (43.5)	291,507 (26.6)	
Heavy	653,764 (7.4)	29,097 (2.7)	
Exercise; Yes (%)	4,640,617 (52.3)	481,636 (44.0)	< .0001
Underlying illness (%)			
Hypertension	2,260,085 (25.5)	302,447 (27.6)	< .0001
Dyslipidemia	1,632,304 (18.4)	187,974 (17.2)	< .0001
Diabetes mellitus	755,131 (8.52)	112,363 (10.25)	< .0001
Metabolic syndrome	820,865 (11.27)	273,136 (10.23)	< .0001
Initial laboratory findings ^b^			
Hemoglobin, g/dL	14.29 ± 1.33	11.28 ± 0.99	< .0001
Creatinine,	1.02 ± 0.89	0.93 ± 0.75	< .0001
GFR	88.48 ± 45.14	87.10 ± 34.40	< .0001

BMI, body mass index; GFR, glomerular filtration ratio; No, number.

^a^ Anemia is defined as a hemoglobin level less than 13 g/dL in males and 12g/dL in females according to WHO criteria.

^b^ Mean ± standard deviation.

^c^ Individuals with income less than 20% of total population were identified.

### Risk of IBD in patients with anemia stratified with respect to the hemoglobin level

**Fig 1:**
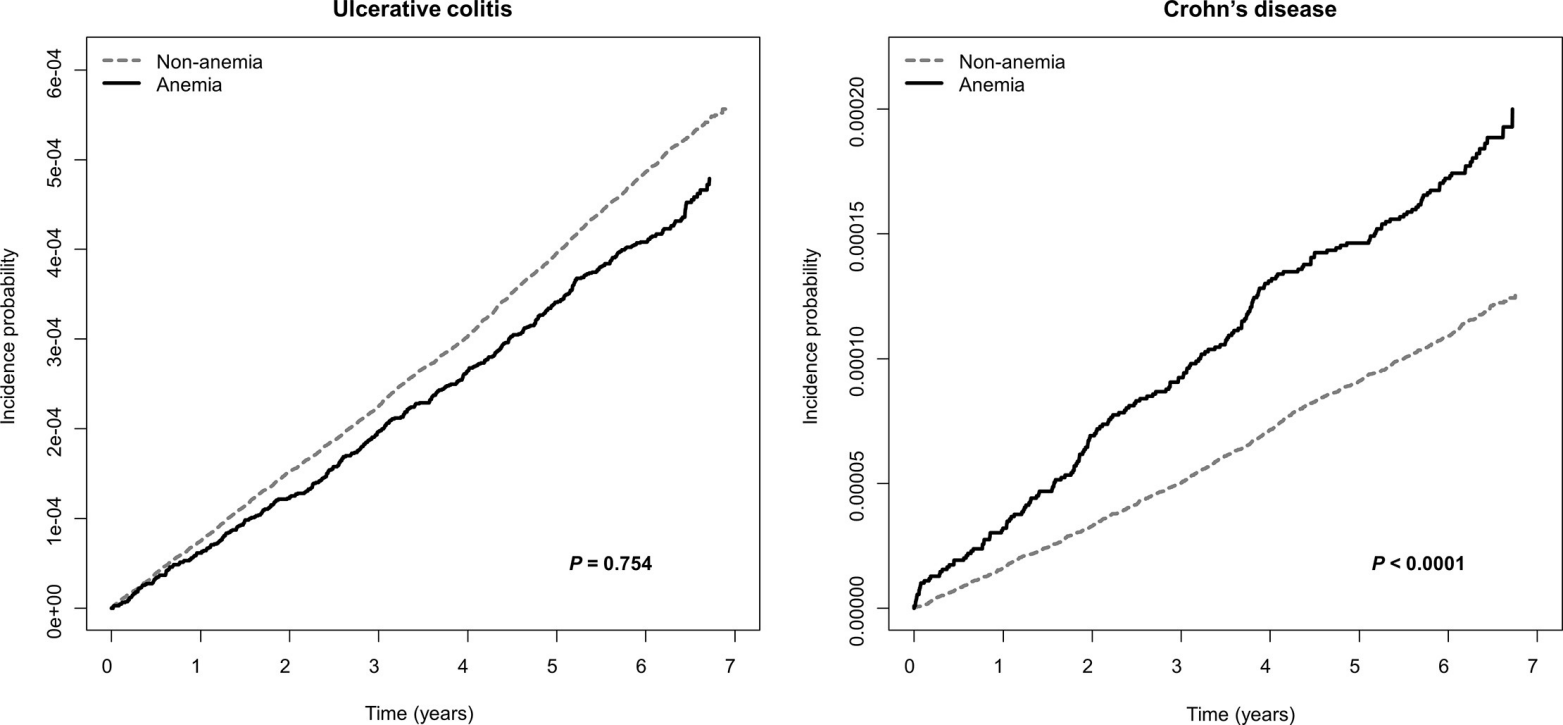
Cumulative incidence probability of ulcerative colitis and Crohn’s disease according to the presence of anemia.

**Fig 2:**
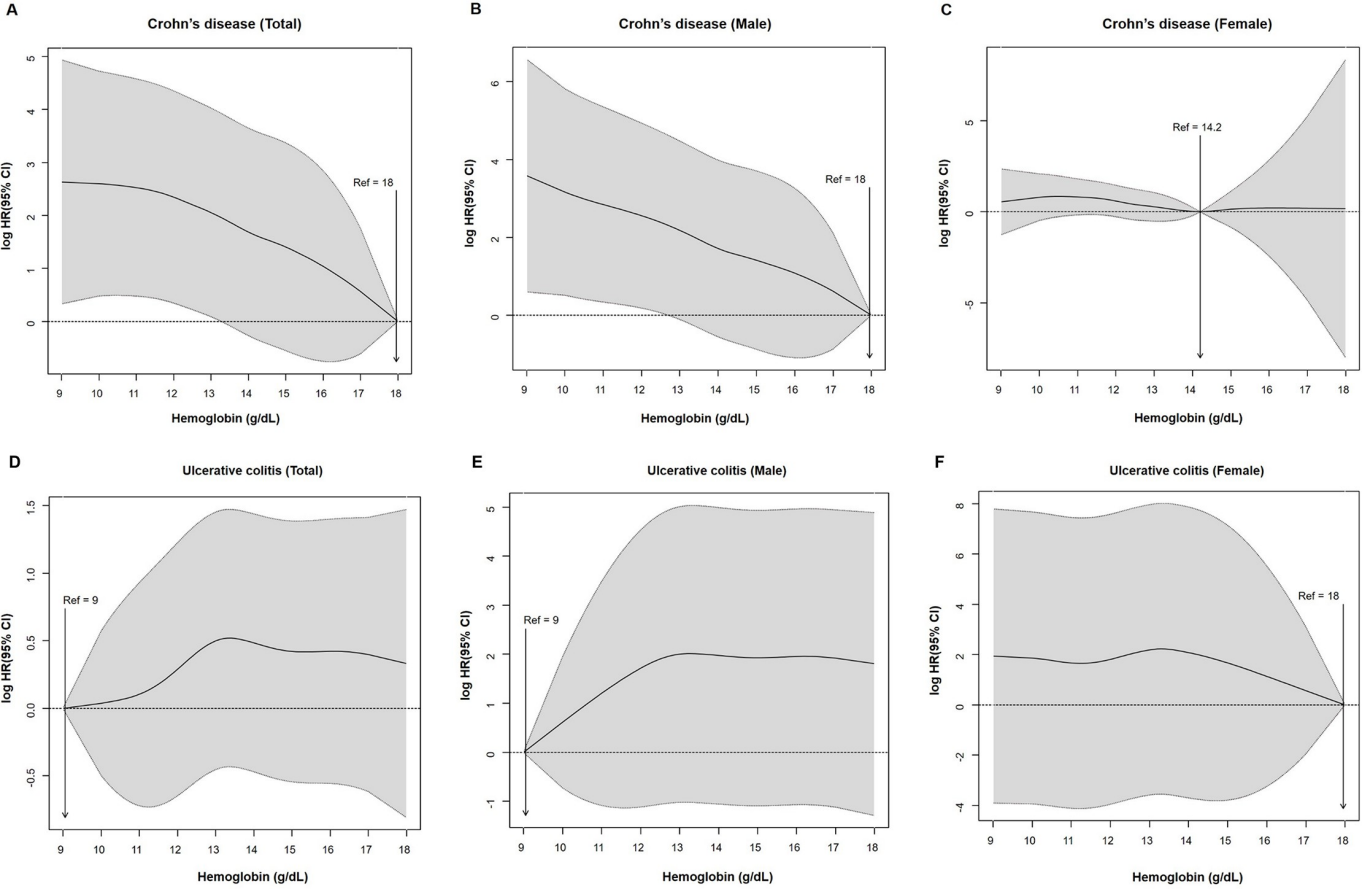
Risk of inflammatory bowel disease according to the hemoglobin levels (A, D) in total population, (B, E) male (C, F) female. CI, confidence interval; HR, hazard ratio; Ref, reference.

We analyzed the incidence and HR of IBD in anemic and non-anemic groups. The incidence (per 100,000 person-years) of CD in participants with anemia was higher than that in non-anemic participants (2.89 vs 1.84; P < 0.0001). Model 1 was adjusted with respect to age and sex in anemic and non-anemic groups, whereas model 2 was adjusted for age, sex, BMI, smoking, alcohol consumption, physical activity, income, and GFR. The HR of CD was significantly higher in the anemic group than the non-anemic group as analyzed using both models 1 and 2 (model 2; adjusted HR, 2.084; 95% CI, 1.769–2.455; *P* < 0.001; Table 2). The incidence (per 100,000 person-years) of UC in the anemic and non-anemic groups was 6.88 and 8.11, respectively. The adjusted HR of UC did not significantly differ between anemic and non-anemic groups (model 2, adjusted HR 0.972, 95% CI 0.880–1.073, *P* = 0.754). The cumulative incidence probability of UC and CD was shown in Fig 1. The risk of CD was inversely proportional to the patient hemoglobin level (Fig 2A). There were sex differences in the association between hemoglobin level and the risk of developing CD, as males had stronger associations than females (Fig 2B and 2C). By contrast, there was no significant association between the hemoglobin level and the risk of developing UC (Fig 2D–2F).

**Table 2:** Risk of inflammatory bowel disease according to the presence of anemia.

Anemia	UC (No)	Adjusted HR (95% CI)		CD (No)	Adjusted HR (95% CI)	
		Model 1 ^a^	Model 2 ^b^		Model 1 ^a^	Model 2 ^b^
Yes	467	1.033 (0.936–1.140)	0.972 (0.880–1.073)	196	2.243 (1.904–2.641)	2.084 (1.769–2.455)
No	4,533	1 (Ref.)	1 (Ref.)	1,030	1 (Ref.)	1 (Ref.)
*P*-valve		0.0702	0.7594		< .0001	< .0001

CD, Crohn’s disease; CI, confidence intervals; HR, hazard ratios; No, number; Ref., reference; UC, ulcerative colitis.

^a^ Model 1: Adjusted by age and sex.

^b^ Model 2: Adjusted by age, sex, body mass index, smoking, alcohol consumption, exercise, metabolic syndrome, income, and glomerular filtration ratio.

### Anemia during follow-up and the risk of developing IBD

Among 6,696,826 individuals without anemia at the index date, 352,464 (5.3%) patients developed anemia at the two-year follow-up exam. The risk of developing CD was significantly higher in anemic patients than in individuals without anemia in 2011 (adjusted HR 2.763, 95% CI 2.254–3.387) (Table 3). The risk of developing CD was more prominent in male patients with post-anemia than in female patients (adjusted HR, 4.311 vs. 2.178, respectively). By contrast, there was no significant difference in the risk of UC development based on the presence of post-anemia among non-anemic patients at baseline (adjusted HR 1.135, 95% CI 1.005–1.282), regardless of sex.

**Table 3:** Incidence and risk of inflammatory bowel disease according to the presence of anemia during follow-up among non-anemic individuals.

		Total			Male			Female		
	Anemia during follow-up ^a^	Event (No)	Incidence (/100,000 person-year)	HR (95% CI) ^b^	Event (No)	Incidence (/100,000 person-year)	HR (95% CI) ^b^	Event (No)	Incidence (/100,000 person-year)	HR (95% CI) ^b^
UC	No	2,891	8.91	1 (ref.)	2087	10.74	1 (ref.)	804	6.19	1 (ref.)
	Yes	310	8.84	1.135 (1.005,1.282)	121	13.71	1.144 (0.946,1.383)	189	7.20	1.124 (0.959,1.318)
CD	No	583	1.80	1 (ref.)	423	2.18	1 (ref.)	160	1.23	1 (ref.)
	Yes	133	3.79	**2.763 (2.254,3.387)**	60	6.80	**4.311 (3.225,5.762)**	73	2.78	**2.178 (1.649,2.876)**

CD, Crohn’s disease; CI, confidence intervals; HR, hazard ratios; No, number; Ref., reference; UC, ulcerative colitis.

^a^ Anemia during follow-up was defined as a case that became anemic in the 2-year follow-up exam among non-anemic individuals at baseline.

^b^ HR was adjusted by age, sex, body mass index, smoking, alcohol consumption, exercise, metabolic syndrome, income, and glomerular filtration ratio.

### Subgroup analysis

**Fig 3:**
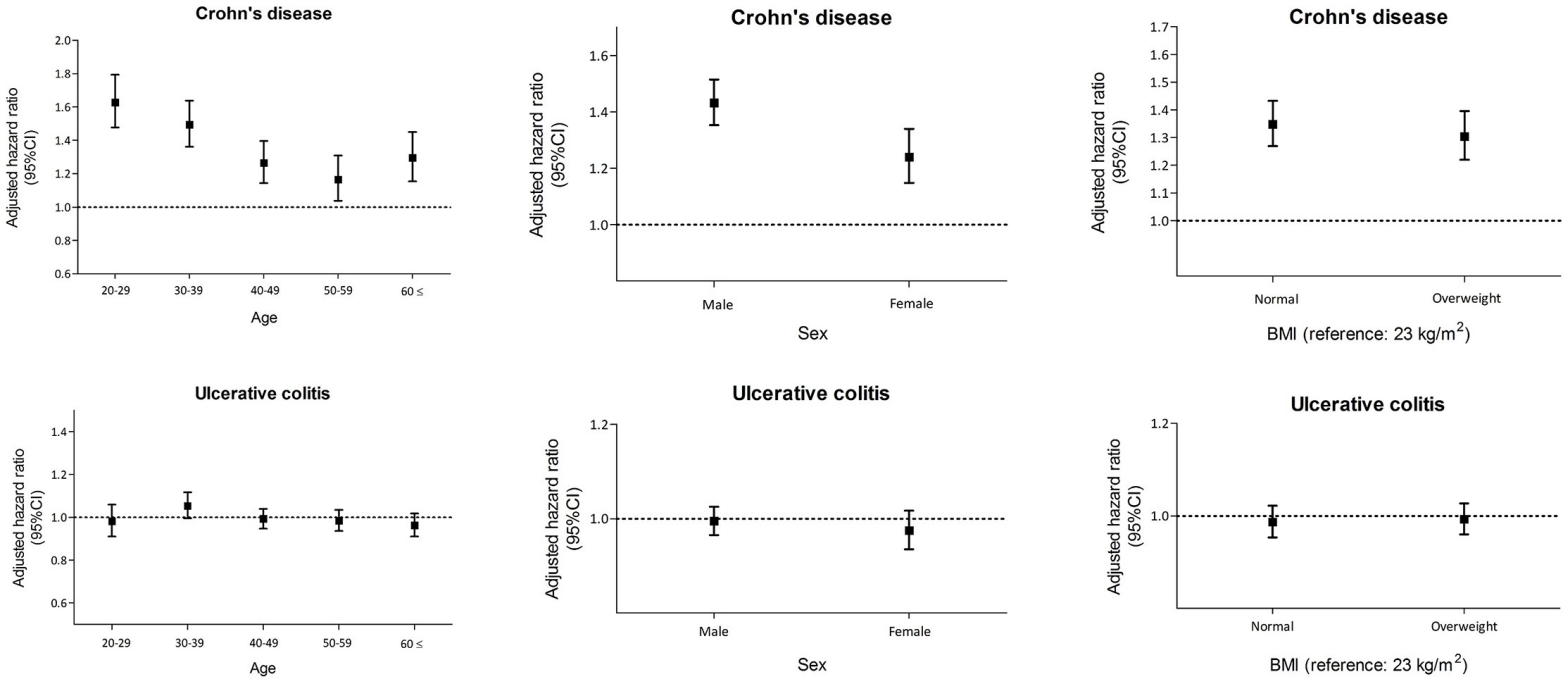
Hazard ratio of inflammatory bowel disease in patients with anemia according to age, sex, and body mass index. Error bars represent 95% CI. CI, confidence interval.

Subgroup analyses were performed according to age, sex, CKD, overweight, smoking behavior, and alcohol consumption to assess the impact of anemia on the risk of developing IBD (Fig 3). A J-curve relationship was observed between age and the risk of CD in anemic patients. By contrast, anemic patients did not have a significantly increased risk of developing UC compared to the non-anemic group, regardless of age. Male patients with anemia had a significantly higher risk of CD development than female patients (adjusted HR, 1.432 vs. 1.240, respectively; *P* = 0.0136). persons with anemia and BMI under 23 kg/m^2^ also had a higher risk of CD than overweight patients (adjusted HR, 1.348 vs. 1.304, respectively; *P* = 0.0041). CKD, smoking behavior, and alcohol consumption did not affect the risk of developing CD in anemic patients (Table 4). The risk of developing UC in anemic patients was similar to that in the non-anemic group regardless of this variants. Alcohol consumption is known to increase iron uptake, and can possibly confound the association between anemia and development of UC and CD. HR of CD was 2.038 (1.723–2.411) in anemic patients who did not drink alcohol and 3.482 (1.686–7.191) in anemic patients who drank alcohol, which was not statistically significant (*P-value* for interaction, 0.182). Therefore, the effect of anemia on CD development was independent of alcohol consumption (S1 Table).

**Table 4:** Subgroup analysis of risk for inflammatory bowel diseases among anemic patients.

Subgroup		UC	HR (95%CI)	CD	HR (95%CI)	*P*-value for Interaction (CD)
Age	20–29		0.982 (0.911,1.059)		1.628 (1.478,1.794)	0.0739
	30–39		1.054 (0.996,1.117)		1.494 (1.362,1.639)	
	40–49		0.993 (0.947,1.040)		1.265 (1.144,1.397)	
	50–59		0.984 (0.936,1.035)		1.166 (1.038,1.309)	
	≥ 60		0.963 (0.911,1.018)		1.295 (1.155,1.451)	
Sex	Male		0.995 (0.965,1.025)		1.432 (1.353,1.515)	**0.0136**
	Female		0.975 (0.935,1.017)		1.240 (1.148,1.340)	
CKD	No		0.992 (0.968,1.018)		1.329 (1.268,1.393)	0.5351
	Yes		0.943 (0.853,1.043)		1.295 (1.099,1.526)	
Overweight	No		0.987 (0.953,1.022)		1.348 (1.269,1.433)	**0.0041**
	Yes		0.993 (0.960,1.027)		1.304 (1.220,1.395)	
Smoking	No		0.993 (0.965,1.021)		1.309 (1.241,1.382)	0.383
	Yes		0.971 (0.925,1.019)		1.383 (1.273,1.502)	
Alcohol	No		0.991 (0.966,1.016)		1.321 (1.261,1.385)	0.4965
	Yes		0.957 (0.872,1.052)		1.411 (1.189,1.675)	

CD, Crohn’s disease; CI, confidence intervals; CKD, chronic kidney disease; HR, hazard ratios; UC, ulcerative colitis.

## Discussion

We investigated the incidence and risk of IBD in anemic patients using a large-scale, population-based cohort of nearly 10 million Korean patients. The incidence and risk of CD were significantly higher in the anemic group than the non-anemic group, but this association was not observed for UC. The risk of CD development was correlated with the severity of anemia as determined by the hemoglobin level. When patients were stratified with respect to hemoglobin levels, the risk of CD was 3.3 times higher in the lowest 10% group than in the highest 10% group. The presence of anemia within 2 years was associated with a 2.8-fold increased risk of developing CD compared to the non-anemic group. To the best of our knowledge, this is the first epidemiological study to demonstrate the association between anemia and the development of IBD in the general population.

In a recent study, the median duration from the first IBD symptoms to diagnosis was 6.2 and 2.4 months in patients with CD and UC, respectively. However, 25% of the study population had longer diagnosis time intervals of more than 21 and 6 months, respectively, from the presentation of initial symptoms [18]. The diagnostic delay was associated with increased risk of surgery related to IBD. Considering the time interval between the presentation of intestinal inflammation and symptoms, it is imperative to have a good marker for early detection of IBD to prevent disease-related complications and surgery. Our data indicate that anemia is a promising marker that predicts the presence of chronic intestinal inflammation, especially in CD patients. Thus, patients with recently presented anemia are recommended for further evaluation of the possibility of CD, particularly in light of the impact of newly developed anemia on the risk of CD among individuals without baseline anemia in this study.

Anemia is one of the most common extra-intestinal manifestations related to IBD. Previous studies reported the prevalence and risk factors for anemia in IBD. A large-scale cohort study in the United States reported that the prevalence of anemia in IBD patients was up to 50.1% at the time of diagnosis [19, 20]. An increased risk of anemia was associated with surgery for IBD, high inflammatory marker levels such as C-reactive protein, high erythrocyte sedimentation rate, female sex, and use of biological or immunomodulator therapies [19]. In a European population-based inception cohort study, anemia was more frequent in CD (49%) than in UC (39%) during the first year after diagnosis, and extensive UC and colonic CD with penetrating type were risk factors of anemia development [21]. Another population-based cohort study in Sweden revealed that the extensive colitis of UC and stricturing phenotype of CD were significantly associated with an increased risk of anemia [22]. Patients with IBD and anemia have a poorer prognosis than IBD patients without anemia. Anemia can affect multiple factors in IBD patients, including hospitalization, quality of life, surgical complications including poor outcome, medical costs, and mortality [23–25]. These findings suggest that anemia may present during the early stage of IBD, perhaps on a subclinical level, and may be strongly associated with IBD disease severity and extent, eventually leading to poor clinical outcomes. Diagnostic delays were more prominent for CD than UC, and anemia was more prevalent in CD than UC. Our data suggest that the development of anemia is a marker for preclinical IBD. UC may have fewer chances of developing anemia than CD due to the relatively short diagnostic delay and low prevalence of anemia.

Anemia in IBD is caused primarily by iron deficiency or anemia of chronic diseases [22, 24, 26, 27]. Iron deficiency secondary to gastrointestinal blood loss or chronic inflammatory mucosal damage can cause anemia in IBD. Severe anemia could be caused by chronic mucosal inflammation or microscopic gastrointestinal bleeding that occurs before the diagnosis of IBD. Chronic mucosal inflammation in IBD reduces iron absorption and leads to iron deficiency [28]. Anemia in IBD also can be caused by anemia of chronic diseases with persistent inflammatory status in IBD. Malnutrition and malabsorption via inflamed gastrointestinal tract could cause anemia. A recent study reported that subclinical inflammation may affect iron status and hemoglobin concentrations [29]. However, it remains unclear whether the predictive role of anemia in the detection of CD differs according to the etiology of anemia.

We stratified patients with respect to age to evaluate age-specific risks of CD among anemic patients. The results showed a unique J-shape curve between age and the risk of developing CD. This curve is consistent with the results of a recent Korean population-based cohort study of the incidence of CD with respect to age [30]. These data indicate that the age-specific incidence rates of CD peaked in the teens and twenties, then decreased markedly, and subsequently peaked again in the 60s. A recent Swiss IBD cohort study also reported that pediatric-onset CD was associated with higher rates of anemia compared to adult-onset CD [31]. Taken together, the trends in the age-specific risk of developing CD among anemic patients indicate that anemia is a good marker for early detection of CD in the general population.

The risks of developing CD were more prominent in men than in women in our study. Men with the lowest 10% of hemoglobin levels had a 4.5-fold increased risk of CD compared with the highest 10% group. Considering the high prevalence of anemia in premenopausal women, the incidence of IBD in women is considered to be lower than that in men, which suggests that the presence of anemia for early detection of CD is more critical in men in clinical practice.

This population-based study has several limitations due to its retrospective cohort design. First, we could not assess the etiology of anemia from the claims data. The impact of anemia on IBD extent and severity could not be evaluated. A subsequent population-based study will be required to determine the effects of anemia and anemia type on the development of IBD. Second, there was a concern about immortal time bias because the hemoglobin levels are time-varying covariates. In this retrospective study, serial results of hemoglobin levels within 2 years in the study population were not available, and not all the subjects underwent the 2-year follow-up screening exams. Because the Cox proportional hazard models were established in the subpopulations who participated the 2-year follow-up screening exam among the non-anemic individuals at baseline, time-dependent Cox proportional hazard models were not used for controlling immortal time bias. It should be noted that the presence of anemia identified in a single test at baseline and newly developed anemia at follow-up are useful markers for subclinical CD, respectively.

In conclusion, patients with anemia, especially newly developed anemia, were associated with an increased risk of developing CD, not UC. The risk of developing CD was inversely proportional to the hemoglobin level, which was a marker for anemia severity. A J-curve relationship was observed between age and the risk of developing CD among anemic patients, similar to that observed for the incidence of CD based on age. Therefore, anemia should promote further investigation for early detection of CD in general population, especially in males.

## Supporting information

S1 Table Alcohol consumption and the risk for inflammatory bowel diseases. (DOCX)
