# Supplementary material for: Anemia is associated with the risk of Crohn’s disease, not ulcerative colitis: A nationwide population-based cohort study
Source: PLoS One. 2020 Sep 8;15(9):e0238244. doi: 10.1371/journal.pone.0238244 (PMC7478647; doi:10.1371/journal.pone.0238244)
Supplement: S1 Table — (DOCX) [file pone.0238244.s001.docx]

**S1 Table. Alcohol consumption and the risk for inflammatory bowel diseases.**

| **Alcohol consumption** | **Anemia** | **Total number** | **UC (event, n)** | **HR (95%CI)** | ***P*-value for**  **Interaction** |
| --- | --- | --- | --- | --- | --- |
| No | No | 8,212,376 | 4,201 | 1 (Ref.) | 0.3045 |
|  | Yes | 1,066,827 | 457 | 0.979 (0.886,1.083) |  |
| Yes | No | 653,764 | 332 | 1 (Ref.) |  |
|  | Yes | 29,097 | 10 | 0.719 (0.380,1.360) |  |
| **Alcohol consumption** | **Anemia** | **Total number** | **CD (event, n)** | **HR (95%CI)** | ***P*-value for**  **Interaction** |
| No | No | 8,212,376 | 953 | 1 (Ref.) | 0.1820 |
|  | Yes | 1,066,827 | 187 | 2.038 (1.723,2.411) |  |
| Yes | No | 653,764 | 77 | 1 (Ref.) |  |
|  | Yes | 29,097 | 9 | 3.482 (1.686,7.191) |  |

CD, Crohn’s disease; CI, confidence intervals; HR, hazard ratios; n, number; UC, ulcerative colitis.
